# Safety and Chronic Atrial Lesion Formation With a Large-Tip, Contact-Force-Sensing Multipolar Pulsed Field Ablation Catheter: Effect of Application Number in a Porcine Beating Heart Model

**DOI:** 10.1161/CIRCEP.125.014473

**Published:** 2026-04-01

**Authors:** Luigi Di Biase, Jacopo Marazzato, Marco Schiavone, Fengwei Zou, Taylor Spangler, Rahul Bhardwaj, Jonathan Hsu, Assaf Govari, Andreas Altman, Kevin Okarski, Tushar Sharma, Paras Parikh, Christopher Beeckler, Terrence Troutman, Qi Chen, Hiroshi Nakagawa, William Maddox, Devi Nair, Sanghamitra Mohanty, Vincenzo Mirco La Fazia, Domingo Ynoa, Aung Lin, Andrea Natale, Xiaodong Zhang

**Affiliations:** 1 Department of Cardiology, Montefiore Medical Center, Bronx, NY (L.D.B., J.M., F.Z., D.Y., A.L., X.Z.).; 2 Electrophysiology and Cardiac Pacing Unit, Humanitas Mater Domini, Castellanza, Varese, Italy (J.M.).; 3 Bayside Preclinical Services Inc, Dixon (T. Spangler).; 4 Loma Linda University Heart Institute (R.B.).; 5 University of California, San Diego (J.H.).; 6 Biosense Webster, Irvine, CA (A.G., A.A., K.O., T. Sharma, P.P., C.B., T.T., Q.C., W.M.).; 7 Section of Cardiac Electrophysiology & Pacing, Robert and Suzanne Tomsich Department of Cardiovascular Medicine, Cleveland Clinic, OH (H.N.).; 8 Department of Electrophysiology, St. Bernard’s Heart &Vascular Center, Jonesboro, AR (D.N.).; 9 Texas Cardiac Arrhythmia Institute, Austin (S.M., V.M.L.F., A.N.).; 10 Monzino Hospital Milan, Milano, Italy (M.S.).; 11 Biosense Webster, Yokne’am, Israel (A.G., A.A., K.O., T. Sharma, P.P., C.B., T.T., Q.C., W.M.).

**Keywords:** catheter ablation, electroporation, pulmonary vein, safety, swine

## Abstract

**BACKGROUND:**

Integration of catheter–tissue contact force with pulsed field ablation (PFA) dosing is essential for achieving safe and durable lesion formation with contact force sensing catheters in ventricular models. However, data on the use of these catheters for atrial ablation remain limited. This study evaluated the procedural safety and 30-day lesion characteristics of atrial PFA delivered with the OMNYPULSE ablation catheter in a porcine model.

**METHODS:**

Twelve pigs were randomized to receive either 6 (×6 or Group A) or 12 (×12 or group B) PFA applications per ablation. Following 3-dimensional electroanatomic mapping, PFA was delivered to the right superior and inferior pulmonary veins, cavotricuspid isthmus, right atrial posterior wall, left atrial roof, and mitral annulus. Procedural safety was assessed acutely, and lesion characteristics were evaluated on 30-day histology. Maximum transmurality extent (MTE) was defined as >80% of the lesion span exhibiting continuous endocardial-to-epicardial fibrosis within representative histological sections.

**RESULTS:**

Adequate catheter–tissue contact was achieved during PFA delivery (contact force, 28±15 g). No acute procedural complications were observed. At 30 days, PVs and right atrial sites (ie, cavotricuspid isthmus and right atrial posterior wall) demonstrated near-complete MTE regardless of PFA dosing. In contrast, MTE was significantly lower at the mitral annulus and LA roof compared with other sites (44±30% and 21±26%, respectively; *P*<0.001). Increasing the number of PFA applications (×12) resulted in a remarkable improvement in MTE at these locations, with MTE increases of up to 68% compared with ×6 dosing at these sites.

**CONCLUSIONS:**

Atrial PFA using the OMNYPULSE catheter was feasible and safe in a porcine model. However, lesion transmurality varied by atrial location and was dose-dependent at select sites, underscoring the need for site-specific PFA dosing strategies. Further studies are warranted to define optimal PFA parameters for consistent atrial lesion formation.

**What is Known?**

- Pulsed field ablation (PFA) is a minimally thermal energy source with high myocardial selectivity, leading to well-defined myocardial lesions on histology.
- Although contact force showed to be paramount in achieving adequate lesion formation in the ventricles, the role of catheter-tissue contact is less clear at the atrial level.

**What the Study Adds**

- This study aimed to explore the acute and 30-day feasibility of a multipolar, contact force sensing, ablation catheter delivering PFA on different atrial targets in swine randomized into 2 prespecified PFA dosing regimens.
- Provided that adequate contact force was warranted, both dose regimens proved safe. However, lesion transmurality varied by atrial location and was dose-dependent at select sites, underscoring the need for site-specific PFA dosing strategies.
- Further studies are warranted to define optimal PFA parameters for consistent atrial lesion formation.

As shown in preclinical^1^ and clinical studies^2^ conducted in the setting of radiofrequency ablation, the achievement of adequate catheter-tissue contact and lesion contiguity proved paramount for durable and effective myocardial lesion formation. However, the inadvertent collateral injury to anatomic structures adjacent to the ablated myocardium does represent one of the major limitations of the resistive heating developed by radiofrequency ablations.

Pulsed field (PF) ablation (PFA) is a novel, minimally thermal energy source that displays high selectivity for the myocardial tissue.^3^ PFA implements high voltage and low-energy pulsed waveforms that have been proven to lead to myocardial irreversible electroporation and membrane cell disruption, causing apoptosis and, finally, myocardial cell death.

In the setting of PFA, accruing evidence^4,5^ demonstrates that the synergistic integration of the catheter-tissue contact with the number of PF applications enables predictable, deep, and contiguous PFA lesions in ventricular heart models using basket-like,^6^ ring-like,^7^ and linear tip^8^ catheters. However, preclinical experience with contact force (CF)-sensing catheters for atrial PFA is limited.^9^

In this study, we evaluated the acute procedural and 30-day safety of atrial PFA performed with the OMNYPULSE ablation catheter integrated with the TRUPULSE generator in both right and left atrial (LA) chambers. Additionally, PFA lesion characteristics and endocardial-to-epicardial extent of lesion formation were assessed at 30 days using systematic histological analysis of the targeted atrial sites.

## Methods

The data that support the findings of this study are available from the corresponding author on reasonable request.

### Population and Ablation Tools

The experimental protocol was approved by the Committee on the Use and Care of Animals. The porcine model is regularly used for preclinical testing for electrophysiology cardiac ablations due to the relative similarities to the human cardiac anatomy. The investigated animals were considered following acclimation and prescreen health assessment.

Simulating an EP procedure in a swine beating heart model, 13 swine—6 randomized to group A (nominal PFA dose or ×6 applications per ablation) and 7 to group B (high PFA dose or ×12 applications per ablation)—underwent PFA at predefined atrial targets using the CF-sensing OMNYPULSE ablation catheter (Biosense Webster) and the TRUPULSE generator (Biosense Webster). The features of the ablation system were already described elsewhere.^6^ In brief, the OMNYPULSE is a multielectrode, CF-sensing catheter with a distally located spherical cage implementing 12 electrodes through which PFA is delivered. The system also provides CF evaluation through the deformation of the spring in the CF sensor located proximally to the basket, which allows for measurement of the net magnitude force on the entire basket.^6^

### Mapping and Ablation Modality

Before the procedure, all swine were anesthetized with isoflurane and ventilated mechanically. Percutaneous or surgical access of the right femoral vein was performed. A 3-D shell of the atrial chambers was performed with the PENTARAY catheter or the OMNYPULSE catheter under 3-dimensional mapping guidance (CARTO3 system, Biosense Webster Inc) with the help of an intracardiac ultrasound system. Access to the LA was achieved through trans-septal catheterization. For LA mapping, heparin (10 000–12 000 IU) was administered intravenously with additional doses, as necessary to maintain an activated clotting time >300 s for the duration of the procedure. Intracardiac echocardiography was used to monitor any potential procedure-related complications as well.

PFA was delivered as trains of high voltage bipolar biphasic energy with an irrigation of 4 mL/min and 40 mL/min at dwell and during ablation, respectively. Energy delivery was not gated to the ECG.

According to the randomization scheme, ×6 or ×12 applications per ablation were delivered at different atrial locations, as follows: right atrial posterior wall (RAPW), cavotricuspid isthmus (CTI), right superior and right pulmonary veins, mitral isthmus or mitral annular region (MA), and LA roof.

The GLP study (Good Laboratory Practice) was primarily designed as a safety study to evaluate differences in ablation outcomes between 6× and 12× applications, with comprehensive histological analysis of the atria. At each ablation target—right pulmonary veins, RAPW, CTI, MA, and the LA roof—a series of PF applications (either 6× or 12×) was delivered according to protocol, with a mean CF >25 g (range, 5–50 g), as previously validated by our study group in a ventricular setting.^6^ PF applications were delivered with an interlesion distance <9 mm at PV and RAPW sites. At the remaining locations (CTI, MA, and LA roof), 2 to 3 discrete applications were delivered at sites separated by >12 mm, where anatomic space permitted.

**Figure 1.**
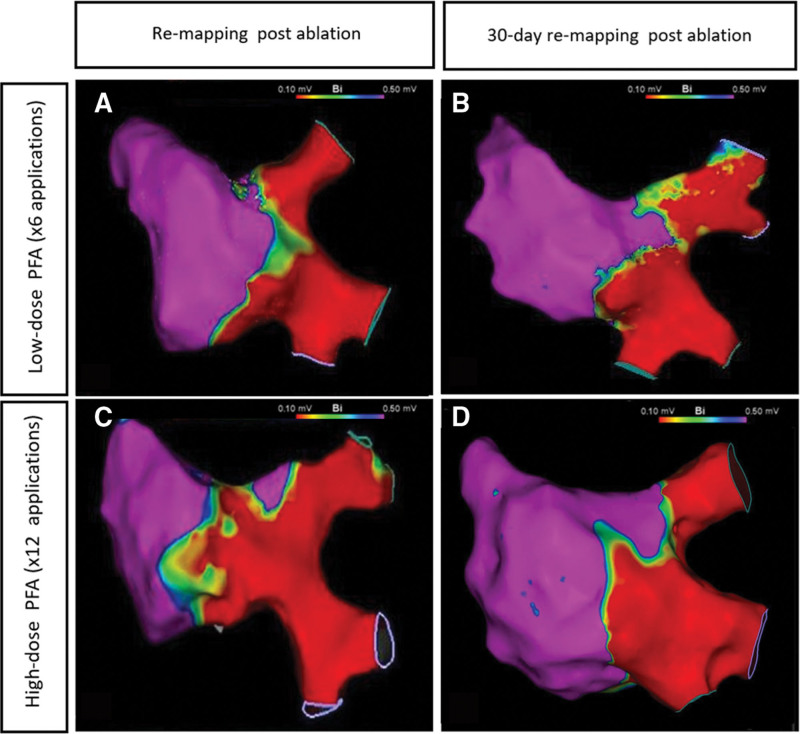
**Postprocedure and 30-day evaluation of pulsed field lesion delivered to pulmonary veins. A** and **C**, Acute and (**B** and **D**) chronic isolation of right pulmonary veins obtained with both low-dose (**A** and **B**) and high-dose (**C** and **D**) pulsed field ablation (PFA), as observed on bipolar voltage maps on the CARTO system. PFA indicates pulsed field ablation.

**Figure 2.**
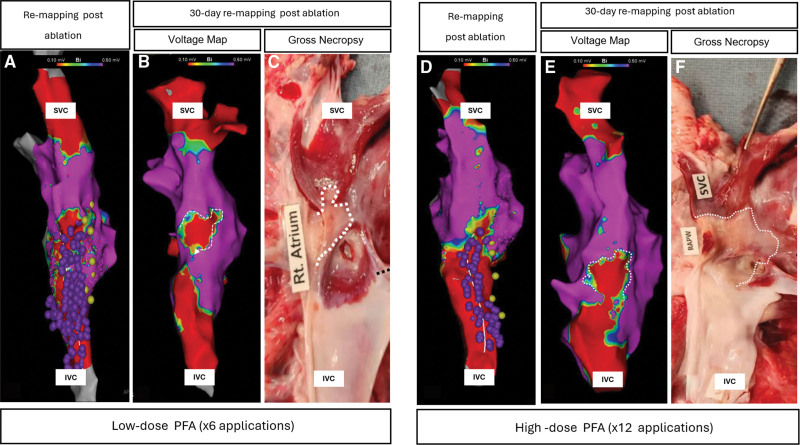
**Postprocedure and 30-day voltage maps and corresponding gross necroscopic evaluation of pulsed field lesions in the right atrial posterior wall (RAPW). A** and **D**, Postprocedure and (**B** and **E**) 30-day voltage maps, with corresponding gross necroscopic evaluation (**C** and **F**), of pulsed field lesions in the right atrial posterior wall for the investigated dosing regimens. Acute and chronic assessments of lesion contiguity are shown for both low-dose (**A–C**) and high-dose (**D–F**) pulsed field ablation (PFA), as observed on bipolar voltage maps acquired with the CARTO system. Corresponding chronic scar formation is demonstrated on gross necropsy and highlighted by dashed white lines (**C** and **F**). In **A** and **D**, solid purple circles represent ablation tags, while yellow tags indicate the course of the right phrenic nerve. Per study design, the ablation line connecting the superior vena cava (SVC) to the inferior vena cava (IVC) was intentionally incomplete (ie, lesions delivered to the inferior portion of the SVC–IVC line) to prevent sinus node injury.

PV isolation (Figure 1) and evaluation of lesion contiguity at RAPW (Figure 2) were both appraised post procedure and 30 days post ablation, followed by histology. The other atrial targets (ie, CTI, MA, and LA roof) were assessed at chronic remapping at 28±2 days post ablation for confirmation of EGM attenuation, followed by histological assessment.

### Chronic Histopathologic Evaluation

Animals were euthanized 30 days postablation, followed by necropsy and histological evaluation. For the 30-day safety assessment, gross and microscopic pathology evaluation focused on both treatment sites and nontarget organs to detect any potential treatment-related damage. A complete necropsy examination was performed on all animals. Gross evaluation included both targeted treatment sites and nontarget organs across cranial, thoracic, and abdominal cavities. After gross examination, tissues were collected, fixed in formalin, and trimmed for histological processing.

Right superior pulmonary vein sites were trimmed in transverse cross-sections to capture the ablation zone, initially at ≈5 to 10 mm intervals, followed by 1 mm serial sections as needed for full lesion representation. Different from the right superior pulmonary veins, right inferior pulmonary veins (RIPVs) were evaluated by multiple radial sections to assess treatment effect because of the peculiar right inferior pulmonary vein anatomy, potentially preventing the correct histological evaluation with clean cross-sections. Most non-PV treatment sites were trimmed in longitudinal serial sections, depending on anatomic geometry and lesion configuration. Tissues were embedded in paraffin and stained with hematoxylin and eosin and elastin–Masson trichrome. Nontarget tissues were stained with hematoxylin and eosin only.

All slides were scanned using an Aperio whole-slide imaging system, and histological evaluation was performed using Aperio ImageScope with integrated digital morphometry tools. The study pathologist was blinded to treatment assignment during gross evaluations and tissue harvest.

Local hallmarks for mechanical injury were sought out, such as steam pop, intracardiac tissue damage, stenosis, or thrombus formation. Lesion features at the targeted sites (neointima thickness, inflammation severity, mineralization, necrosis, and evidence of vessel and nerve sparing) were appraised as well. Finally, nontarget tissues were appraised for evidence of potential unintended effects in downstream organs such as collateral damage or peripheral thromboembolism.

Histological assessment of lesion transmurality was performed on multiple sections obtained from each anatomic treatment site. Sections were generated based on gross identification and orientation of the treatment zone with trimming strategies tailored to anatomic geometry, as described above.

**Figure 3.**
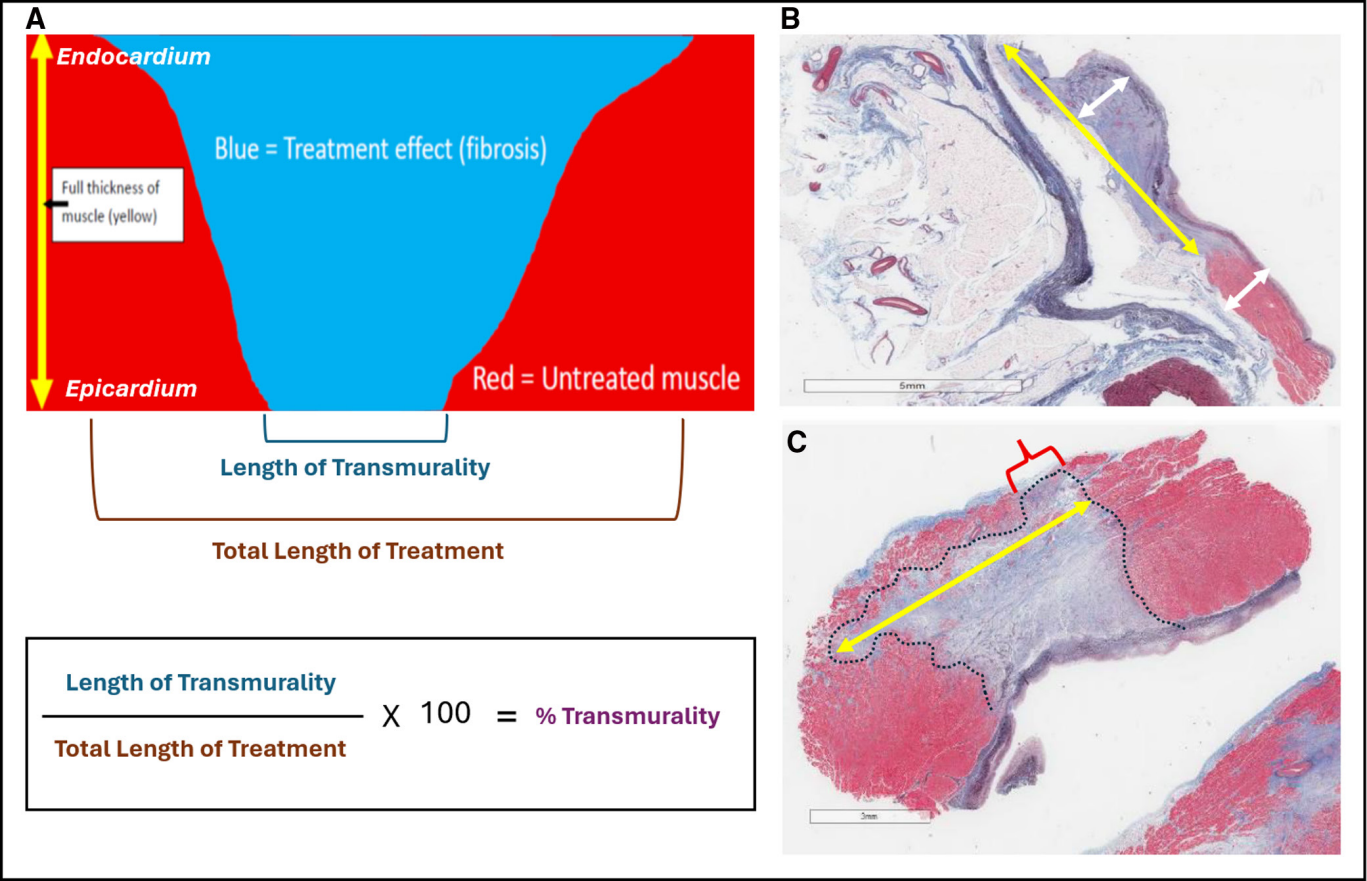
**Histological evaluation of lesion transmurality extent at targeted sites. A**, Schematic illustrating how transmurality extent was quantified as the percentage of lesion span within a single histological section demonstrating full-thickness (endo-epicardial) fibrosis. **B**, Representative longitudinal (radial) histological section through the right inferior pulmonary vein (RIPV), where blue staining indicates fibrotic tissue and red staining indicates viable myocardium; the yellow double arrow denotes lesion length and white arrows indicate myocardial wall thickness. This section demonstrates full transmurality (100%). **C**, Representative histological section from the left atrial (LA) roof treatment site (outlined in black), where only a limited segment (red bracket) demonstrates full-thickness fibrosis, corresponding to an estimated transmurality extent of ≈10%.

For sections judged to adequately represent the treatment zone (representative sections), transmurality was quantified as the percentage of the lesion span demonstrating full-thickness fibrosis from endocardium to epicardium (Figure 3A through 3C). Sections showing a clear absence of treatment effect or incomplete sampling of the treatment zone were classified as nonrepresentative and excluded from quantitative analysis.

Since section-level measurements reflect different sampling planes rather than independent biological replicates, PFA lesion extent was summarized at the anatomic site level using the maximum transmural extent (MTE) observed among evaluated sections for each site in each animal. Lesions with >80% of the measured span showing full-thickness fibrosis within a representative section were classified as transmural under the predefined study criteria.

### Study End Points

Intergroup comparisons were conducted to determine the effect of the 2 PFA dosing regimens (×6 versus ×12 application per ablation) on acute procedural safety and 30-day safety outcomes, including local myocardial injury and collateral or downstream organ damage. In parallel, chronic lesion characteristics and MTE were evaluated at each ablation site per treatment arm.

### Statistical Analysis

Continuous variables were presented as mean±SD for normally distributed variables. Median with range (minimum to maximum) was also presented for non-normally distributed variables. Differences in proportions between groups were tested using the χ^2^ test. Mean values of variables were compared by ANOVA for subgroup analysis when appropriate. Analyses were performed using a significance level of α=0.05 (2-sided). Statistical analyses were performed using the MedCalc software (version 20.115).

## Results

### Postprocedural and 30-Day Safety

Except for one animal, all swine survived the index procedure. In the deceased animal randomized to high PFA dose (group B), histopathologic analysis showed areas of necroscopic contraction band necrosis of cardiac myocytes close to the atrio-ventricular nodal region and likely due to inadvertent atrio-ventricular node ablation leading to sudden cardiac death. The animal was excluded from analysis, thus leading to 6 swine appraised in Group B. No other procedural complication was observed during PFA ablation in both groups.

On 30-day gross and microscopic evaluation of collateral and downstream organs, neither phrenic nerve nor esophageal injury was observed between groups. Both phrenic nerves were thoroughly evaluated, grossly and microscopically, and were determined to be normal in all animals. Moreover, no evidence of thromboembolism or downstream infarction to downstream organs, such as the brain, lung, liver, and kidney were recorded. Gross appraisal of the heart excluded cardiac trauma, mechanical injury, or mural thrombus.

**Figure 4.**
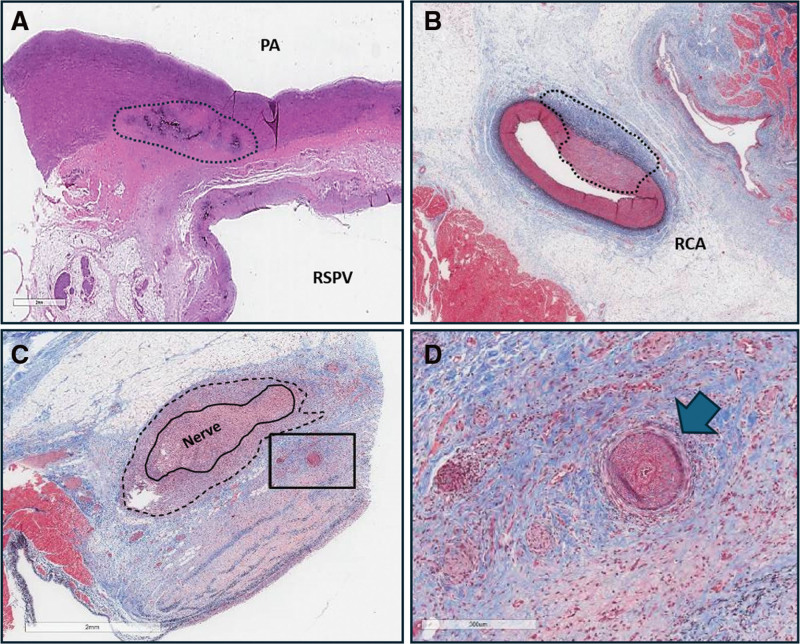
**Pulsed field ablation (PFA)–related minor histological effects on adjacent anatomic structures. A**, Pulmonary artery (PA), (**B** and **D**) vessels, and (**C**) nerves adjacent to atrial myocardium. **A**, Right superior pulmonary vein (RSPV) ablation site (low-dose PFA) demonstrating mild mineralization (black outline) in the outer PA layer without significant intimal changes. **B**, Cavotricuspid isthmus (CTI) ablation site (low-dose PFA) showing minimal arterial wall thickening with limited intimal hyperplasia (black outline). **C**, Right inferior pulmonary vein (RIPV) region (high-dose PFA) demonstrating fibrous myocardial replacement with mild perineural fibrosis (black outlines) and small arteriole fibrosis (boxed). **D**, Higher magnification of the boxed region in **C** showing arteriolar fibrosis (arrow), consistent with localized microvascular injury without associated ischemic injury or clinical sequelae.

As for microscopic evaluation of treatment sites, PFA lesions were predominantly confined to cardiac muscle and tended to spare adjacent fat or other tissues independent of the ablation dose and the target site. Collateral adjacent injuries were, in fact, absent in 87% of cases (89% and 84% in group A and group B, respectively) and were classified as minimal or mild in severity and without observed clinical sequelae during the study period. Adjacent to the right superior pulmonary veins, microscopic and nonsignificant scar tissue was identified in the pulmonary artery in 7 sites (group A=3, group B=4). As observed in Figure 4A, the scar was characterized histologically as small areas of adventitial or mural fibrosis partially extending into the tunica media without affecting the intimal surface of the pulmonary artery. Vessel sparing was observed in most histological sections as well. However, small arterial scars were identified at various treatment sites in 10 different animals (group A=4, group B=6; Figure 4B and 4D). These microscopic vascular scars were predominantly characterized by the presence of fibrotic change around the adventitia or within the intima, and most were morphologically consistent with a vasospastic-type arteriolar injury, a pattern previously described in porcine models and sometimes referred to as segmental arteriomediolysis.^10^ However, none of these microscopic features led to ischemic damage of the appraised myocardium or to any clinical consequences in the investigated swine, nor VF or ST-elevation was detected during ablation. Finally, mild, microscopic, and nonsignificant fibrosis around small nerves in the immediate vicinity of the treated areas was rarely identified at various treatment sites in 7 different animals (group A=3, group B=4), characterized by the presence of perineural fibrosis with a lesser amount of inflammation (Figure 4C)

### Acute Efficacy and Evaluation of 30-Day Maximum Transmurality Extent Per Ablation Site

All PVs were acutely isolated through first-pass isolation, and no signs of PV stenosis were observed. No differences were detected between groups (Figure 1). Similar observations apply to RAPW ablation (Figure 2). EGM attenuation was recorded at the other ablation targets at 30-day re-mapping.

Table 1 shows the number of applications delivered, the count of histological sections, the MTE achieved, and the average CF values during ablation for each ablation target stratified per randomized ablation arm.

**Table 1.**
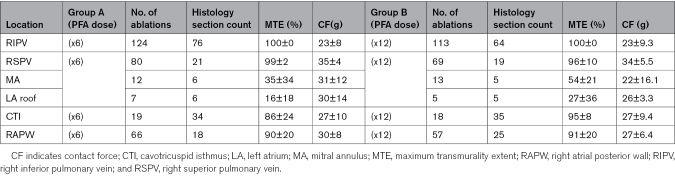
Number of Applications Delivered, Count of Histological Sections, Maximum Transmurality Extent, and the Average Contact-Force Values During Pulsed Field Ablation for the Randomized Dose Regimens and the Appraised Atrial Ablation Targets

As shown in Table 1, a total of 563 applications (318 in group A and 245 in group B) were delivered to the investigated targets. Most applications were performed at right pulmonary vein sites (64% versus 74%), followed by RAPW (21% versus 23%), CTI (6% versus 7%), MA (4% versus 5%), and finally the LA roof (2% versus 2%) for low-dose and high-dose PFA, respectively.

On 30-day histopathologic analysis, 118 out of 435 histological sections (27%) were regarded as nonrepresentative histological sections and therefore, excluded from analysis. Three-hundred and 17 (73%) histological sections were considered: 161 (51%) in group A and 156 (50%) in group B.

In the representative sections, PFA lesions were characterized as stable, quiescent, fibrovascular scar tissue with absent to minimal or mild inflammation, and minimal to mild mineralization. Minimal necrosis and thrombosis were rarely observed, the latter in the form of irrelevant small platelet clumps. Neointima thickness was a frequent finding at treatment sites and averaged <400 µm with no difference recorded between groups.

**Figure 5.**
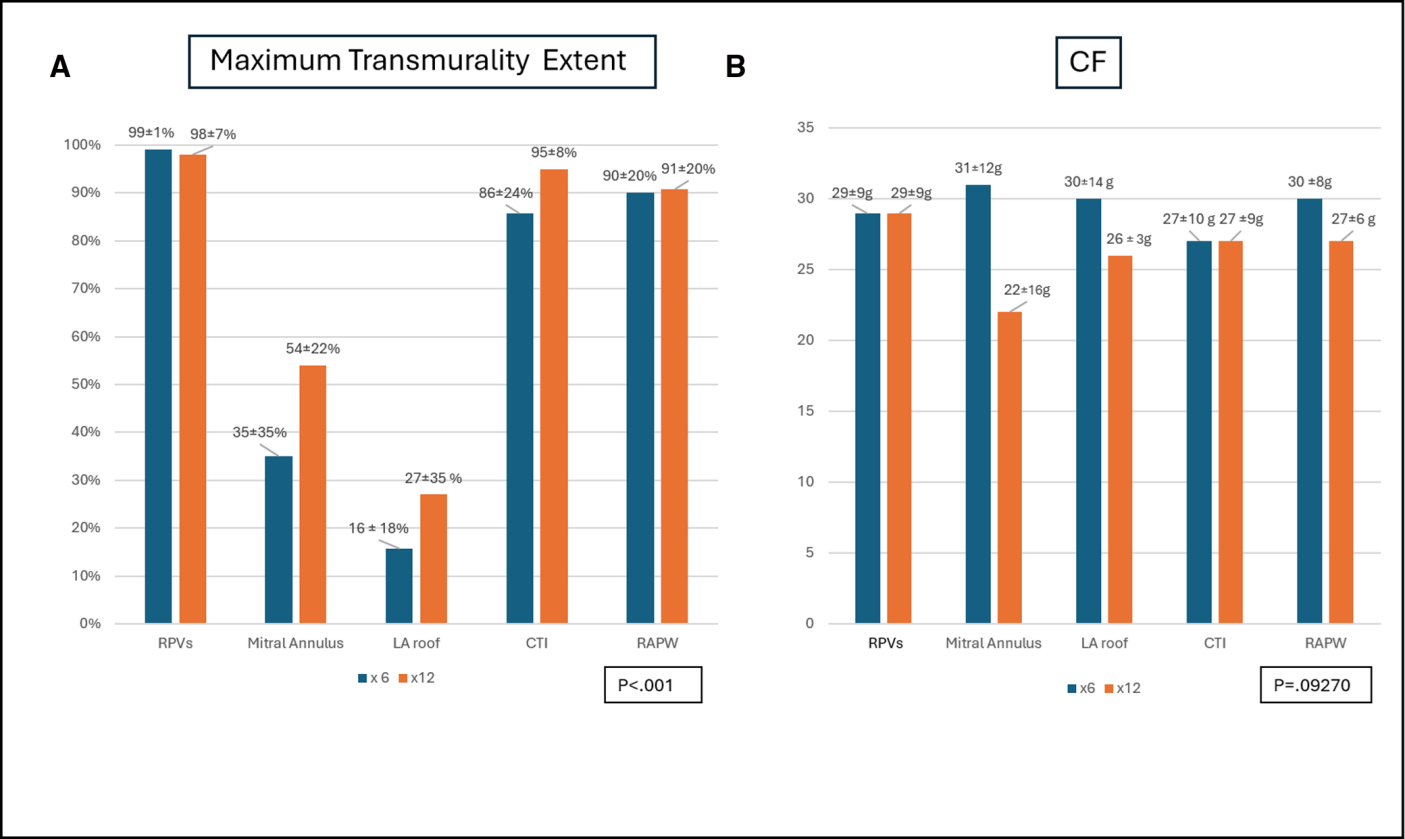
**Analysis of maximum transmurality extent on 30-day histology and the average catheter-tissue contact during ablation. A**, Analysis of maximum transmurality extent on 30-day histology and (**B**) the average catheter–tissue contact (CF) during ablation for each anatomic target per randomized pulsed field ablation dosing regimen. CTI indicates cavotricuspid isthmus; LA, left atrium; RAPW, right atrial posterior wall; and RPV, right pulmonary veins.

Although mean MTE was similar between the 2 investigated ablation protocols (71±38% versus 79±32% for ×6 versus ×12 applications per ablation, respectively, *P*=0.330), the maximum extent of full-thickness endo-epicardial fibrosis (MTE) differed by anatomic target (*P*<0.001; Figure 5A).

**Figure 6.**
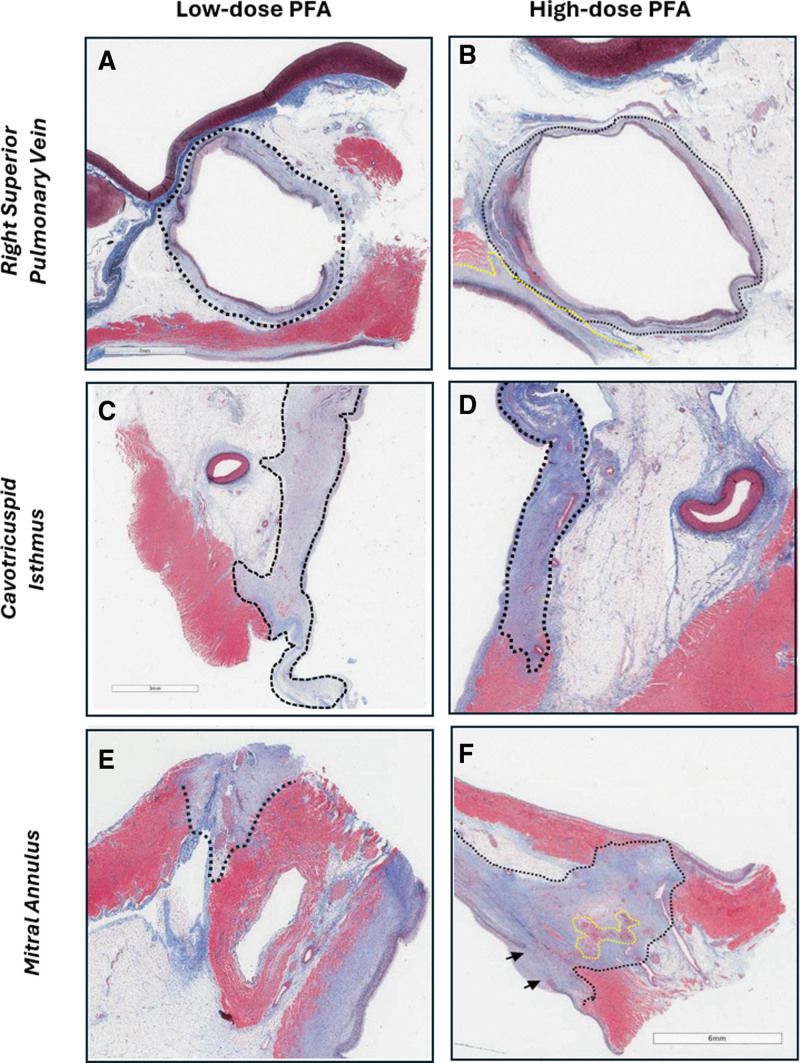
**Histological sections at selected anatomic targets 30 days after pulsed field ablation (PFA). A** and **B**, Right superior pulmonary vein (RSPV), (**C** and **D**) cavotricuspid isthmus (CTI), and (**E** and **F**) mitral annulus (MA) obtained 30 days after low- and high-dose PFA. Across both dosing groups, pulmonary vein targets (**A** and **B**) and right atrial sites including the CTI (**C** and **D**) frequently exhibited near-complete maximum transmurality extent (>80%). In contrast, lower transmurality extent was generally observed at the mitral annulus and left atrial roof. Representative sections from the higher-dose group (**F**) demonstrated greater transmurality compared with low-dose PFA (**E**), potentially reflecting increased application overlap.

PVs and right atrial targets displayed remarkably high MTE across both ablation dose groups (right pulmonary veins, 99±5%; CTI, 90±18%; and RAPW, 90±19%; Figure 6A through 6D). Conversely, the maximum endo-epicardial lesion extent was significantly lower for MA and LA roof locations (44±30% and 21±26% for MA and LA roof, respectively), with some evaluated sections reporting low or absent transmural extent (as low as 0% MTE) at these sites (Table 2). However, compared with low-dose PFA, a greater number of applications per ablation (×12) was associated with a higher probability of identifying endo-epicardial full-thickness injury in representative sections, with a higher—albeit nonsignificant—MTE at both MA (35±35% for low-dose versus 54±22% for high-dose PFA; *P*=0.318) and LA roof (16±18% versus 27±36% for ×6 and ×12 applications per ablation, respectively, *P*=0.516; Figures 5A, 6E, and 6F).

**Table 2.**
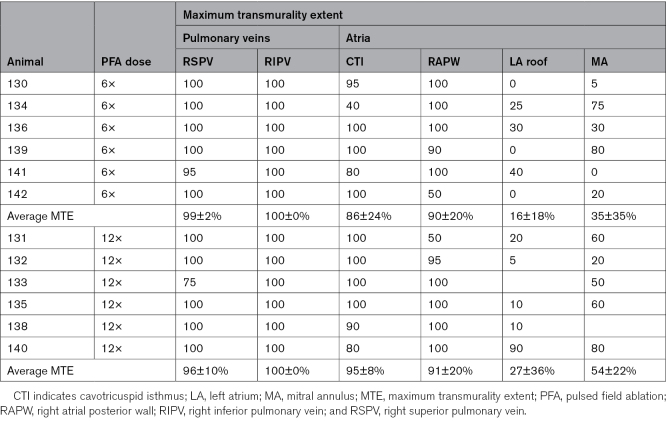
Maximum Transmurality Extent in the Investigated Animal Per Ablation Dose Regimen and Atrial Target

During ablation, the average catheter-tissue contact warranted by OMNYPULSE was remarkable (28±15 g; range, 5–50 g) with no difference between groups (29±15 g versus 29±15 g for low-dose and high-dose PFA, respectively; *P*=0.2257) even when stratified per different CF range (CF <15 g: 50% versus 50%; [CF, 15–30 g]: 52% versus 48%, and CF >30 g: 54% versus 46%, for group A and group B, respectively; *P*=0.4287) and anatomic targets (*P*=0.9270; Figure 5B).

## Discussion

PFA is a novel and minimally thermal energy source that exploits the biophysics of irreversible electroporation to cause cell death. A variety of PFA ablation parameters, including the number of pulses, catheter tip orientation, and specific PFA workflows, proved to act in synergy with adequate catheter-tissue contact (CF) in achieving wide and deep ablation lesions during PFA delivery.^4^ However, the experience with CF-sensing catheters is limited, especially on atrial targets, including nonpulmonary vein targets.

In this randomized, preclinical study, the CF-sensing OMNYPULSE ablation catheter proved safe during PFA delivery to a variety of left and right atrial targets, showing mature chronic lesions with nerve and vessel sparing as observed on 30-day histology in most cases. No significant safety issues were observed at adjacent and downstream organs.

Histologically, no features of thermal ablation injury or clinically significant collateral tissue damage were identified at the investigated ablation doses. Minor, localized vascular changes were infrequently observed, including minimal pulmonary artery fibrosis and small-vessel alterations consistent with a vasospastic-type arteriolar injury, without associated ischemic or clinical consequences. The limited pulmonary artery involvement observed may be related to anatomic factors at the treatment sites. Histological evaluation identified only minimal small-vessel changes in treated areas, without evidence of associated clinical effects in the evaluated animals. Notably, the microvascular fibrosis and focal arteriomedial changes observed in this study were rare, mild in severity, and limited to small intramural arterioles within the ablated zone. These findings are most consistent with localized vascular responses to tissue injury, such as treatment-associated endothelial disruption or vasoreactivity, rather than classic segmental arterial mediolysis as described in human pathology.^11,12^ Importantly, no associated thrombosis, downstream ischemia, or organ infarction attributable to these microscopic vascular changes was identified, and no related clinical sequelae were observed during the 30-day survival period. To date, no published human studies have reported segmental arterial mediolysis following PFA, further limiting the translational relevance of this entity in the current context.

As for chronic lesion formation, preclinical studies assessing the relationship between catheter-tissue contact and lesion depth in a swine ventricular model suggest that the higher the contact during PFA, the deeper the lesion created^13–16^ with both basket-like^6^ and linear^8^ CF-sensing catheters showing a synergistic effect between CF and PFA dose.^6^

However, data on the combined effect of CF and PFA dose on atrial lesion size are scarce.

Investigating a proprietary linear catheter with customizable energy settings, Hua *et al* showed that PFA delivered to right atrial targets—superior vena cava, CTI, right superior pulmonary vein, and RIPV—led to chronic transmural fibrosis as evaluated on 30-day histology.^14^ Likewise, in an experimental setting built up with the integration of the linear SmartTouch catheter and the TRUPULSE generator, Hsu et al^17^ reported durable and transmural lesions at the PV location regardless of the PFA energy used. Nevertheless, LA roof, posterior wall, and atrial appendage location relied on PFA dose to achieve full-depth lesions. The combined role of CF and PFA dose delivered to both atria was first explored by Di Biase et al^9^ in a randomized study where the authors proved that adequate CF maintenance during ablation (13.9±4.1 g on average) led to similar chronic histology features across the investigated randomized arms (low-dose, high-dose PFA, and RF ablation). However, unlike PV location, where lesion transmurality was easily achieved independently of the energy source utilized, full-depth lesions were not observed at the MA and LA roof, even when the system was toggled to high PFA energy dose during ablation.

Although variability in atrial endocardial–epicardial thickness may partly account for these findings,^18^ accurate characterization of ablation lesions across atrial regions requires histological assessment that accounts for site-specific anatomy. Given the substantial geometric heterogeneity among the PVs, CTI, RAPW, MA, and LA roof, site-specific histological sectioning centered on the lesion core is necessary to evaluate the extent of PFA-induced endocardial–epicardial fibrosis. Of note, none of the previously published studies^9,14,17^ have applied a consistent or explicitly defined histopathologic framework.

To our knowledge, this is the first randomized study to evaluate the safety of a CF-sensing, basket-like PFA catheter for atrial ablation beyond the PVs, and the first to appraise the impact of the number of PFA applications on 30-day atrial MTE at each ablation target investigated using clear, predefined site-specific histopathologic criteria.

In this study, histological assessment demonstrated that the maximum extent of full-thickness, endo-epicardial fibrosis (MTE) identified in representative sections capturing the lesion core differed primarily by anatomic target. Pulmonary vein and right atrial targets most frequently exhibited high MTE (ie, >80% per prespecified study criteria) within at least one well-oriented section across both dosing arms, whereas lower MTE was observed at the MA and LA roof. At these latter sites, some evaluated sections demonstrated shallow or nontransmural injury, particularly in the lower-dose (×6) group.

Although mean catheter–tissue CF was high and comparable across anatomic targets and dosing arms (28±15 g), a higher number of applications per ablation (×12) was associated with a greater likelihood of identifying full endo-epicardial thickness injury in representative sections at the MA and LA roof, reflected by higher, though not statistically significant, MTE values. These findings suggest that site-specific anatomic factors, and potentially lesion overlap requirements, influence the MTE achievable at certain atrial locations under the conditions studied. As suggested by other studies in the field,^9,14,17^ these findings may be partially attributable to the variable endo-epicardial thickness of the atrial targets investigated. In the swine heart, atrial thickness ranges from <2 mm within the PVs and posterior LA wall to 3 to 6 mm in the LA ridge and LA roof to 2.5 to 8 mm in the MA and up to 10 mm in the CTI.^18^ Accordingly, consistent with anatomic differences, application stacking or increased overlap between ablations during PFA energy delivery may be associated with greater lesion depth and the MTE in thicker atrial substrates such as the MA and LA roof. This behavior contrasts with RF ablation, in which application stacking has primarily been associated with increased local edema without substantially increasing lesion depth.^7,19^

However, these results should be interpreted in the light of several study limitations.

First, this study was originally designed to evaluate the safety of OMNYPULSE and was not powered to detect modest site-specific or dose-dependent associations for areas outside of the PVs, such as the MA and LA roof.

Second, according to the prespecified protocol, only a limited number of applications were delivered in the LA outside of the PVs in discrete locations that were not contiguous. As a result, comparisons across anatomic regions should be interpreted cautiously, as ablation strategies differed by site. Although direct comparisons are therefore limited, a higher application dose was associated with greater MTE at the MA and LA roof. However, findings outside the PVs should not be interpreted as definitive evidence at these anatomic sites, and further study focused on PFA beyond PVs is required in this field.

Third, the variable histology sampling may have caused a biased estimate of the MTE at specific atrial sites. Given the sampling design, these results generally reflect the maximum extent of full-thickness myocardial injury identified within treated zones. In anatomically appropriate transverse sections, this may correspond to circumferential completeness within the sampled plane, whereas at other sites the measurements reflect lesion extent within the plane of section rather than overall lesion continuity or procedural efficacy.

Finally, a wide range of PFA catheters with differing geometries and generator integrations is currently available, resulting in heterogeneous ablation protocols and lesion characteristics. Lesion formation during PFA is, in fact, influenced by pulse number, electric field strength, and the number of electrodes in contact with the tissue. Although it was shown that CF is influenced by the catheter ablation shape^4^ with an increased catheter-tissue contact required for basket-like versus linear-tip PFA catheters,^20,21^ it is still unknown whether specific atrial targets require target-specific CF thresholds to optimize lesion formation.

Future large-scale prospective preclinical and clinical studies are warranted to identify the ablation parameters required to achieve complete endo-epicardial fibrosis with PFA at atrial sites beyond the PVs.

## Conclusions

PFA with the OMNYPULSE ablation catheter integrated with the TRUPULSE generator demonstrated a favorable safety profile for PFA ablation of different atrial targets, including non-PV sites. Under the conditions studied, the investigated dosing regimens were capable of producing full-thickness myocardial injury at atrial treatment sites, with the maximum extent of transmural involvement varying by anatomic location. Thinner atrial structures more frequently exhibited near-complete transmural injury, whereas thicker regions, such as the MA and LA roof, showed lower MTE within representative sections. These findings suggest that atrial wall thickness and site-specific anatomy strongly influence the achievable extent of PFA-induced transmural injury and highlight the potential need for tailored application strategies when targeting thicker atrial substrates.

## Article Information

### Disclosures

Dr Di Biase is a consultant for Stereotaxis, Biosense Webster, Boston Scientific, Abbott Medical, and I-Rhythm and has received speaker honoraria/travel from Medtronic, Atricure, Biotronik, Baylis Medical, and Zoll. Dr Hsu has received honoraria from Medtronic, Abbott, Boston Scientific, Biotronik, Janssen Pharmaceuticals, Bristol-Myers Squibb, Pfizer, Sanofi, Zoll Medical, Altathera, iRhythm, Acutus Medical, Galvanize Therapeutics, viz.AI, and Biosense-Webster, research grants from Biotronik and Biosense-Webster, and has equity interest in Vektor Medical. C. Beeckler and Dr Govari are Biosense Webster Employees. T. Spangler is a consultant for Biosense Webster and provides pathology consulting services to multiple companies developing cardiac pulsed field ablation technologies. The other authors report no conflicts.
